# The Water Extract of Rhubarb Prevents Ischemic Stroke by Regulating Gut Bacteria and Metabolic Pathways

**DOI:** 10.3390/metabo14040216

**Published:** 2024-04-12

**Authors:** Xiaoyao Liu, Yuxi Wang, Yuan Tian, Jiahui Hu, Zhen Liu, Yuncheng Ma, Wenhui Xu, Weiling Wang, Jian Gao, Ting Wang

**Affiliations:** 1 School of Chinese Medicine, Beijing University of Chinese Medicine, Beijing 100029, China; 20210941048@bucm.edu.cn (X.L.); jiahui_hu9@bucm.edu.cn (J.H.); 2 School of Life Sciences, Beijing University of Chinese Medicine, Beijing 100029, China; 20220941092@bucm.edu.cn (Y.W.); 20220931162@bucm.edu.cn (Y.M.); 3 School of Chinese Material Medica, Beijing University of Chinese Medicine, Beijing 100029, China; 20220935165@bucm.edu.cn (Y.T.); 20220935166@bucm.edu.cn (Z.L.); xwh@bucm.edu.cn (W.X.); 4 Beijing Research Institute of Chinese Medicine, Beijing University of Chinese Medicine, Beijing 100029, China

**Keywords:** dahuang (rhubarb, RR), ischemic stroke, gut microbiota, metabolomics, ^1^H-NMR

## Abstract

Rhubarb (RR), Chinese name Dahuang, is commonly used in the treatment of ischemic stroke (IS). However, its potential mechanism is not fully elucidated. This study intended to verify the effect of RR on IS and investigate the possible mechanism of RR in preventing IS. IS in male rats was induced by embolic middle cerebral artery occlusion (MCAO) surgery, and drug administration was applied half an hour before surgery. RR dramatically decreased the neurological deficit scores, the cerebral infarct volume, and the cerebral edema rate, and improved the regional cerebral blood flow (rCBF) and histopathological changes in the brain of MCAO rats. The 16S rRNA analysis showed the harmful microbes such as *Fournierella* and *Bilophila* were decreased, and the beneficial microbes such as *Enterorhabdus*, *Defluviitaleaceae*, *Christensenellaceae*, and *Lachnospira* were significantly increased, after RR pretreatment. ^1^H-nuclear magnetic resonance (^1^H-NMR) was used to detect serum metabolomics, and RR treatment significantly changed the levels of metabolites such as isoleucine, valine, N6-acetyllysine, methionine, 3-aminoisobutyric acid, N, N-dimethylglycine, propylene glycol, trimethylamine N-oxide, myo-inositol, choline, betaine, lactate, glucose, and lipid, and the enrichment analysis of differential metabolites showed that RR may participate in the regulation of amino acid metabolism and energy metabolism. RR exerts the role of anti-IS via regulating gut bacteria and metabolic pathways.

## 1. Introduction

Stroke is a common clinical cerebrovascular disease that has high rates of incidence, recurrence, disability, and mortality, and a high treatment cost, presenting a serious danger to human health [1,2]. Stroke mainly includes ischemic stroke (IS) and hemorrhagic stroke. IS accounts for more than 80% of stroke, and there is still an increasing annual trend with the rapid growth in the aging population [3,4,5]. IS is an acute disease caused by cerebral vascular obstruction, leading to cerebral ischemia, hypoxia, necrosis, and neurological function defects [6,7,8]. The intravenous recombinant tissue plasminogen activator (rt-PA) is the only drug currently approved by the FDA for the therapy of IS [8,9]. However, the use of rt-PA in the clinic is limited due to its strict indications and contraindications [9,10]. More effective strategies for IS are urgently needed.

IS belongs to the category of “stroke” in traditional Chinese medicine (TCM). The history of treating stroke in TCM is long-standing. Rhubarb plants (*Rheum tanguticum* Maxim. ex Balf., *Rheum palmatum* L., or *Rheum officinale* Baill., RR), also known as dahuang in Chinese. The medicinal parts of dahuang, as stipulated in the Chinese Pharmacopoeia, are the roots and rhizomes. As first recorded in Shennong Bencao Jing, it was commonly used in the treatment of stroke based on the theory of Tong Fu in TCM. The mechanism of action and clinical efficacy of the Tong Fu method in the treatment of stroke have been reported, the prescriptions for stroke based on the theory of Tong Fu have also been summarized in the literature, and the results all show that RR is a commonly used drug in the treatment of stroke [11,12,13,14]. As shown in the literature, study results from research related to the treatment of IS based on the theory of Tong Fu show that RR is the most frequently used drug, which shows that RR is a key drug in the treatment of IS using the method of Tong Fu [15]. The curative role of RR for IS has been reported in multiple studies, such as reducing cerebral infarct volume and neurological deficit score [16,17,18]. However, because of the complex pathogenesis of IS, the effect and mechanism of RR on IS treatment have still not been entirely elaborated.

Previous studies have found that RR as a purgative could improve colon mucosal barrier injury and severe acute pancreatitis by regulating gut microbiota [19,20]. The gut flora is a large and complex microbial community that lives in the gastrointestinal tract. It was reported that the gut flora plays a crucial part in keeping the internal balance of the body, which modulates the occurrence and progression of illness. Previous studies suggested that gut microbes could influence metabolism and cerebral function by way of the microbiome–brain–gut axis [21,22,23]. Lee et al. found that fecal transplantation from young mice improved the outcomes of older mice with stroke, which was related to microbes that generate short-chain fatty acids [24]. Hence, we speculated that RR might regulate gut microbes and metabolites and the internal relationship between them, which may affect the brain’s physiological function through the blood circulation or the gut–brain axis.

Metabolites are the downstream products of the regulation of genes and proteins, and therefore more immediately reflect the changes in the physiological or pathological state of the organism, and the mechanism of development of IS is associated with alterations in metabolites [25,26]. A metabolomics study found that the phenylacetylglutamine (PAGIn) in plasma in IS patients was dramatically increased compared with that in healthy controls [27]. Refined Huanglian–Jiedu decoction was revealed to play a neuro-protective effect against IS via modulation of the metabolism of energy, amino acids, and nucleic acids based on metabolomics using ^1^H-nuclear magnetic resonance (^1^H-NMR) [28]. However, whether the disordered composition of gut microbes and levels of metabolites can be reversed by RR treatment in middle-cerebral-artery-occlusion (MCAO) rats is still uncertain.

In our study, we observed the role of RR in MCAO rats and further analyzed its impact on alterations in gut microbiota and serum metabolites using 16S sequencing and ^1^H-NMR non-targeted metabolomics, which laid the experimental foundation for further clarification of the mechanism of RR for IS.

## 2. Materials and Methods

### 2.1. Chemicals and Reagents

The following were used: Hematoxylin and eosin dye liquor (Wuhan Servicebio Technology Co., Ltd., Wuhan, China; No. G1004 and G1002), 2,3,5-triphenyltetrazolium chloride (TTC) stain (Beijing Biodee Biotechnology Co., Ltd., Beijing, China; No. BN20391). The standard substances emodin, chrysophanol, aloe-emodin, physcion, rhein, sennoside A, emodin-8-glucoside, and (+)-catechin (Shanghai Yuanye Biological Co., Ltd., Shanghai, China; No. B20240, No. B20238, No. B20772, No. B20242, No. B20245, No. B20380, No. B20241, No. B21722). Chromatographic grade acetonitrile, methanol, and formic acid (Thermo Fisher Scientific Inc., Santa Clara, CA, USA; NO. A955-4, A456-4 and 28905; Purity > 99%). Extract of Ginkgo Biloba Leaf (EGb761, Dr. Willmar Schwabe GmbH & Co., Karslruhe, Germany; No. 5550918).

### 2.2. Preparation of RR and Dosage Calculation

RR was bought in Beijing Bencao Fangyuan Pharmacy Co., Ltd. (Beijing, China), and identified as *Rheum tanguticum* Maxim. ex Balf. by Xiangri Li, professor of TCM processing at Beijing University of Chinese Medicine. The voucher sample (accession No. 201009) was located at the Beijing Research Institute of Chinese Medicine, Beijing University of Chinese Medicine. To prepare herbal extracts, 1000 g of RR was crushed into a powder using a pulverizer (Zhongcheng Pharmaceutical Machinery, Changsha, China). The herbal powders were decocted 3 times for 20–30 min each decoction, using 10 volumes (*w*/*v*) of distilled H_2_O. Three decoctions were combined and filtered, and the RR dry powder was obtained by concentrating at reduced pressure and freeze-drying. The extract rate of RR was 30%. The equivalent dose for rats was calculated using the conversion ratio between rats and humans based on body surface area (BSA) [25], and the high dose of RR (RR-H) used in rats was calculated using the following formula based on the clinical dosage (see the details in Supplementary Materials):

Dosage of RR-H = 15 g/person/day × 6.2/70 kg

The medium dose of RR (RR-M) is half the dosage of RR-H, while the low dose of RR (RR-L) is a quarter of the dosage of RR-H. The RR powder was dissolved in double-distilled water (ddH_2_O) to appointed concentrations (10, 20, and 40 mg/mL). Thus, the test doses of RR in rats were administered to RR-L (100 mg/kg/d), RR-M (200 mg/kg/d), and RR-H (400 mg/kg/d, the clinical maximum dose) groups.

### 2.3. Chemical Characterization of RR Extract

The chemical components of the RR extract were analyzed using ultra-high-performance liquid chromatography (UHPLC) combined with Q-Exactive Orbitrap mass spectrometry (MS). RR dry powder (1 g) was dissolved with 50 mL of methanol. The chrysophanol, emodin, (+)-catechin, rhein, physcion, aloe-emodin, sennoside A, and emodin-8-glucoside (purity > 97%) were configured with methanol in a mixed standard solution at a concentration of 125 μg/mL. The filtrates of the RR extract and standard solution were placed in a liquid-phase vial after passing through a microporous membrane of 0.22 μm. 

UHPLC analysis was carried out using a Dionex Ultimate 3000 System (Thermo Scientific, Santa Clara, CA, USA). The separation of the test solution and standards was carried out on an AQUITY UPLC HSS T3 column (2.1 mm × 100 mm, 1.8 μm) at 35 °C. The injection volume and flow rate were 5 μL and 0.3 mL/min, respectively. The mobile phases were made up of acetonitrile (A) and 0.1% formic acid/water (*v*/*v*) (B). The gradient elution was performed using the following conditions: 0–50 min (5–90% A), 50–50.1 min (90–5% A), and 50.1–55 min (5–5% A). The Q Exactive Plus Orbitrap MS (Thermo Scientific, Santa Clara, CA, USA) was used to analyze the Electrospray ionization (ESI)-MS. The RR sample was analyzed in both ionization modes. The MS analysis was conducted on the heated electrospray ionization source (HESI) with temperatures of 400 °C and 320 °C for the ion source and the capillary, respectively. The voltages of the ionization source and tube lens were 3.5 KV (+) and 3 KV (−), and 55 V, respectively. Nitrogen (purity > 99.99%) was used as a sheath gas and auxiliary gas at 35 arb and 10 arb flow rates, respectively. 

The full scan was performed using Fourier transform (FT, resolution 70,000, mass scan range of *m*/*z* 120.00–1800.00), and the data-dependent acquisition (ddMS^2^) and the higher-energy collision dissociation (HCD) were used for the acquisition of MS spectra. The raw MS data were processed using the software Xcalibur 2.2.0, and the composition was confirmed based on the retention time, high-resolution accurate mass, and MS^n^ multi-stage fragmentation, combined with the data of the standard, ChemSpider database, and literature reports.

### 2.4. Animals and Experimental Design

#### 2.4.1. Experimental Animals and Ethics Statement

A total of 144 SPF Sprague Dawley (SD) rats (male, body weight 230–260 g) were obtained from SPF Biotechnology Co., Ltd. (Beijing, China, certificate No. SCXK (Beijing) 2019-0010). All animals were administered in a barrier environment animal house for free access to food and water with a 12 h light/dark cycle at 20–25 °C room temperature and 40–70% humidity. Our experimental schemes and all surgical and experimental procedures were endorsed and approved by the Animal Ethics Committee of the Beijing University of Traditional Chinese Medicine (No. BUCM-4-2022111001-4036). All surgical and experimental procedures were designed to avoid or minimize discomfort, distress, and pain to the animals.

After 3 days of acclimatization, 144 rats were randomized into the Sham, Model, RR-L, RR-M, RR-H, and EGb761 groups, with each group containing 24 rats. The MCAO models were performed in all the other groups, while anesthesia and vascular dissection were performed in the Sham group. About 30 min after the drug administration, the rats were anesthetized by intraperitoneal injection of 20% urethane (1000 mg/kg), and fixed supine on the operating table, and the surgery was performed with the suture-occluded method, as previously described [29,30,31]. After surgery, all the rats were laid on a heating cushion at 37 °C until they recovered from anesthesia. Medical intervention was conducted in the RR-L, RR-M, RR-H, and EGb761 groups by gavage with RR extracts (100, 200, and 400 mg/kg), and in EGb761 (40 mg/kg), sequentially at 0.5 h before the MCAO surgery, while equal volumes of drinking water were given to the rats in the Sham and Model groups.

#### 2.4.2. Neurobehavioral Assessment

About 24 h after MCAO, the neuroethology of all animals was assessed by a blinded investigator using a five-level scale (0–4 points) [31], by which neural function and injury degree could be evaluated. The scoring details are as follows: the behavior was completely normal and there was no damage to neural function, which was recorded as 0 points; when the rat was lifted off the ground and the contralateral forelimb was internally rotated, the rat had slight nerve damage, which was marked as 1 point; observing the walking of the rats, it can be seen that the rats circle to the contralateral side, indicating the rats had moderate nerve injury, which was recorded as 2 points; observing the walking of the rats, it can be seen that the rats fall to the contralateral side, indicating the rats have severe nerve function injury, which was marked as 3 points; the rats could not walk and had no consciousness of spontaneous movement, indicating the rats had severe neurological damage, which was scored as 4 points.

#### 2.4.3. Cerebral Edema Rate

After neurological assessment, the rats were anesthetized with 5% isoflurane and then underwent CO_2_ euthanasia, and their brains were quickly removed. The brain tissue was divided into the right and left hemispheres, which were weighed separately. Cerebral edema was assessed by measuring the cerebral edema rate, and the calculation formula is as follows:

Cerebral edema rate (%) = (Weight_ischemic hemisphere_ − Weight_non-ischemic hemisphere_)/(Weight_ischemic hemisphere_ + Weight_non-ischemic hemisphere_) × 100%.

#### 2.4.4. Evaluation of Cerebral Infarction Volume by TTC

At 24 h after the MCAO surgery, the rats were anesthetized with 5% isoflurane and then underwent CO_2_ euthanasia, and their brains were quickly removed. The brain tissue was cut into six consecutive coronal sections with a 2 mm thickness after excluding the olfactory bulb, cerebellum, and lower brain stem. The brain sections were soaked and incubated in a 1% TTC solution at 37 °C for 30 min under the condition of protection from light. After the sections were stained and fixed in 10% neutral formalin fixative for 24 h, the fluid on the surface of the tissues was drained, and the tissues were placed in order and photographed. The cerebral infarct volume was calculated by the Image J software (version 1.54g) and the percentage of cerebral infarct volume = (cerebral infarct volume/whole brain volume) × 100%.

#### 2.4.5. Regional Cerebral Blood Flow (rCBF) Measurements

The rCBF disorder is a golden index for the onset and progression of IS [32]. The Perimed Laser Speckle Imager (Pericam PSI HR; Sweden) was used to measure rCBF at 24 h after MCAO based on Laser Speckle Contrast Analysis technology. The operating distance of the instrument was set at 13.5 cm, the sampling frequency was 21 images/s, and the PIMSoft software (version 1.11.1) was used for monitoring blood flow and processing data. The area below the coronal sutures and within the linear temporalis side was classified as the area of interest, and the following formula was used to calculate the reduced rate of rCBF:

The reduced rate of rCBF (%) = (ROI 2 − ROI 1)/ROI 2 × 100 (%)

where ROI 1 is the monitoring value of rCBF in selected areas on the ischemic side, and ROI 2 is the monitoring value of rCBF in selected areas on the non-ischemic side.

#### 2.4.6. Histopathological Staining

The brain tissue was removed and immediately fixed in 4% paraformaldehyde for more than 24 h after the rats were perfused with the saline and 4% paraformaldehyde at 24 h after MCAO, and the tissue was dehydrated, paraffin-embedded, and sliced into 3 μm thick sections using a tissue slicer (Shanghai Leica Instrument Co., Ltd. RM2016, Shanghai, China). After the sections were dewaxed to water using xylene and gradient ethanol, they were sequentially subjected to hematoxylin staining for 8 min, color separated in 1% hydrochloric acid of alcohol for 30 s, reversed to blue using ammonia for 5 min, and stained with eosin for 2 min. They were then dehydrated with gradient ethanol, made transparent in xylene, and capped with neutral gum.

### 2.5. Gut Microbiota Determination and Data Analysis

The feces of the colon segment of the rats were collected 24 h after MCAO to analyze the intestinal microbial composition. After the total genomic DNA was extracted and quality checked, it was amplified by PCR with specific primers (designed according to the specified sequencing region (V3–V4 variable region of 16S rDNA)), and the amplified products were quantified using a fluorescent quantitative PCR system. The sequencing library was constructed using the kit from Illumina, and sequencing was carried out with the platform provided by Illumina. PE reads from Illumina sequencing were spliced, controlled, and filtered. Species diversity analysis was performed by sample-based OUT clustering analysis. The 16S rRNA database was analyzed by the RDP classifier Bayesian algorithm, and the bioinformatics analysis was performed via the QIIME platform. Further detailed information is available in Supplementary Materials. 

### 2.6. H-NMR Spectroscopy and NMR Data Analysis

The serum of 200 μL obtained from rats 24 h after MCAO was added to the deuterated phosphate buffer of 400 μL, vortexed, mixed, and centrifuged (4 °C, 12,000 r/min) for 20 min, and the supernatant was transferred to an NMR tube of 5 mm for future analysis. A 600 MHZ NMR spectrometer was employed to record the ^1^H-NMR spectra at 599.672 MHz and 298 K. The relevant measurement parameters and data processing before principal component analysis (PCA) and orthogonal partial least squares discriminant analysis were carried out by referring to the literature (OPLS-DA) [33]. The chemical shift of the NMR spectra was corrected by the lactate and was referenced at δ 1.33. The online Human Metabolome Database (HMDB) and relevant published references were employed to identify the metabolites. The integral area of the metabolites was assessed using the R software package (version 4.3.1), the clustering analysis of the samples was performed using PCA to reveal trends between the groups, and the degree of separation between the different groups was visualized through the OPLS-DA. The OPLS-DA models were validated by replacing the response values of the tests and calculating R^2^ (the total explained variance, related to the prediction accuracy of the model) and Q^2^ (the predictive capacity, related to the fitting ability of the model), and the number of model validations was 200. The color-coded coefficient map was produced to visualize and filter the variables that impact the model. The variable importance for projection (VIP) value and *t*-test were used to screen for metabolites, which were considered to be differential metabolites with a *p*-value ≤ 0.05 and VIP > 1. The enrichment analysis was performed by applying the Metaboanalyst platform with differential metabolites, and the KEGG online database was used to analyze the significantly perturbed pathways of the differential metabolites. Further detailed information is available in Supplementary Materials.

### 2.7. Statistical Analysis

Data are expressed as mean ± SD. The Student’s *t*-test for two groups or the one-way ANOVA for multiple groups was conducted, and the significant difference was considered to be *p* < 0.05. The Spearman’s correlation analysis was employed to assess the relevance of the intestinal microbes to the serum metabolites.

## 3. Results

### 3.1. Chemical Compositions of RR

**Figure 1:**
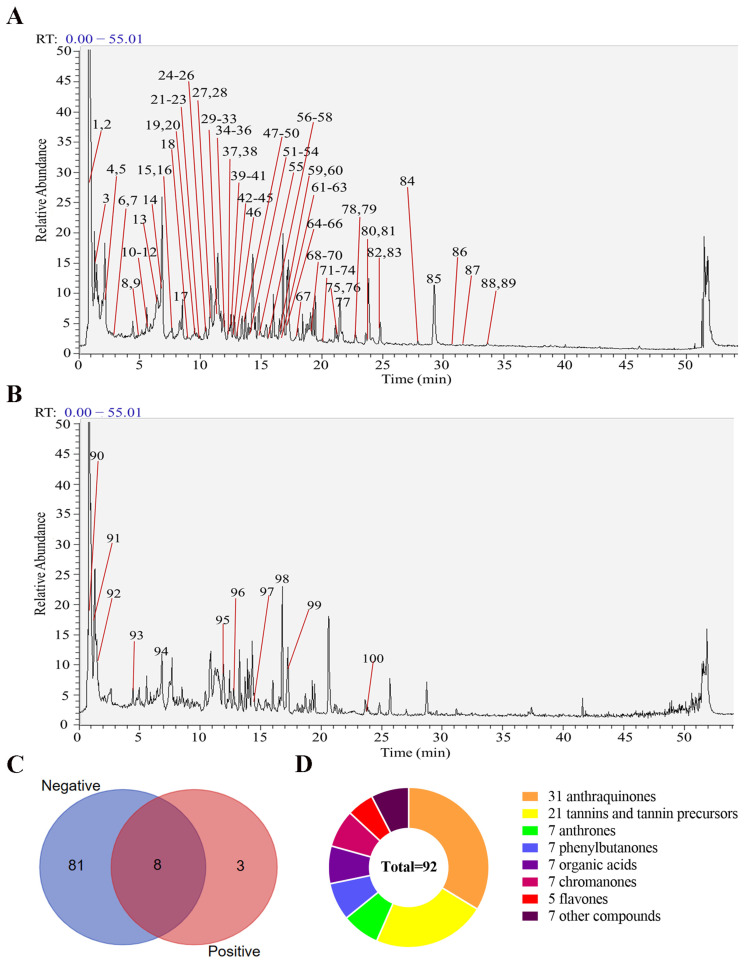
Components of RR were identified by UHPLC-Q-Exactive Orbitrap MS. (**A**) RR in negative ion mode for total ion flow diagram (see Supplementary Table S1 for the details of No. 1–89). (**B**) RR in positive ion mode for total ion flow diagram (see Supplementary Table S1 for the details of No. 90–100). (**C**) Venn diagram of ingredients from positive and negative ion modes. (**D**) Classification of RR chemical components.

A total of 92 chemical components, comprising 31 anthraquinones, 7 anthrones, 21 tannins and tannin precursors, 7 phenylbutanones, 7 organic acids, 7 chromanones, 5 flavones, and 7 other classes of compounds were identified in RR. Among them, there were 11 compounds in the positive ion mode, 89 compounds in the negative ion mode, and 8 compounds both in the positive and negative ion modes, as shown in Figure 1.

### 3.2. RR Pretreatment Significantly Attenuated Cerebral Ischemic Injury

**Figure 2:**
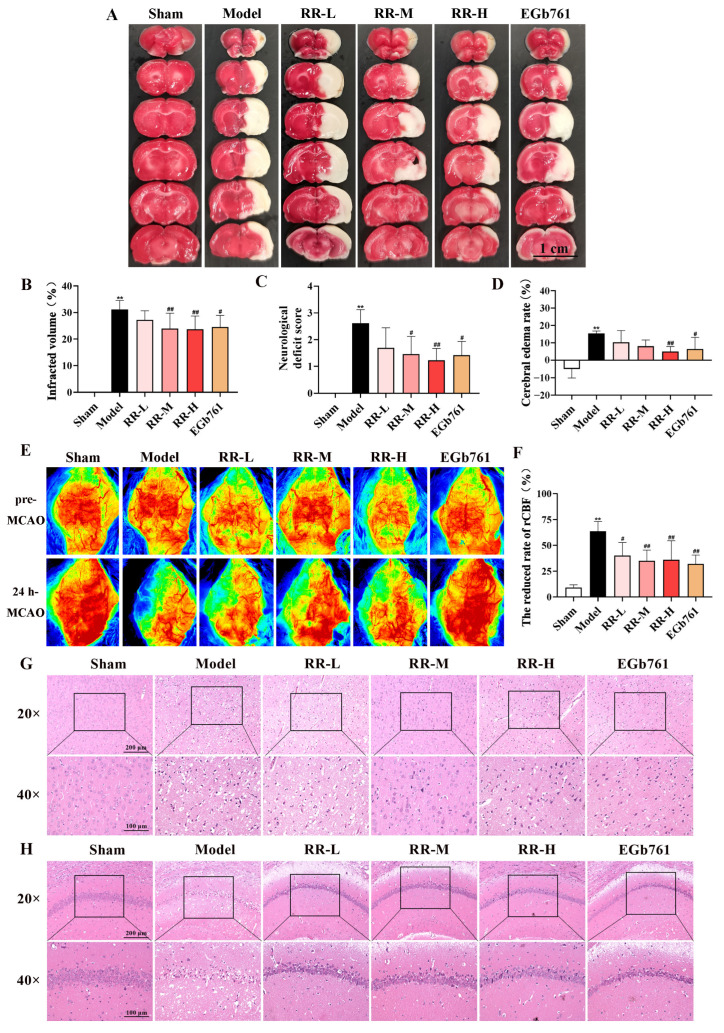
The protective effect of RR on the MCAO model rats. (**A**) Representative images of TTC-stained brain slices are demonstrated. (**B**) Statistical results of infarct volumes in each group. (**C**) Statistical results of the neurological scores in each group. (**D**) Statistical results of cerebral edema rate in each group. (**E**) Representative pictures of rCBF from the same rats of pre-MCAO and 24 h MCAO. (**F**) Statistical results of rCBF in each group. (**G**) Representative pictures of HE staining of the brain cortex. (**H**) Representative pictures of HE staining of the hippocampus CA1 region. Values are expressed with mean ± SD. Versus the Sham group: ** *p* < 0.01. Versus the Model group: ^#^ *p* < 0.05, ^##^ *p* < 0.01.

There was no cerebral infarction occurred in the Sham group, as indicated in Figure 2A,B. The MCAO surgery led to an explicit cerebral infarct in the rats, which decreased after the administration of RR. The infarct volume in the Model group of the rats was dramatically greater compared with that in the Sham group (*p* < 0.01), while it was significantly reduced in the RR-M, RR-H, and EGb761 treatment groups when compared to the Model group (*p* < 0.05 or *p* < 0.01). Additionally, the neurological score of the Model rats was dramatically higher than that of the Sham group (*p* < 0.01), as shown in Figure 2C, indicating that model rats showed a worse neurological condition. Interestingly, the RR-M, RR-H, and EGb761 rats showed a dramatic decrease in neurological scores compared to the Model group (*p* < 0.05 or *p* < 0.01), demonstrating that RR, especially RR-M and RR-H, significantly improved the neurological function of models.

### 3.3. RR Pretreatment Alleviated Brain Edema Induced by MCAO

The brain edema 24 h after MCAO was measured. The cerebral edema rate was significantly increased in the MCAO group (15.45 ± 1.34%, *p* < 0.01) compared to that in the Sham group (−5.06 ± 5.07%), as shown in Figure 2D. EGb761 and RR treatment significantly decreased the cerebral edema rate ((6.38 ± 6.74% in EGb761 group, *p* < 0.05) and (5.02 ± 2.84% in RR-H group, *p* < 0.01)), indicating that pretreatment using RR, especially RR-H, significantly alleviated brain edema induced by MCAO. 

### 3.4. RR Pretreatment Significantly Alleviated the Changes in Regional Cerebral Blood Flow

At 24 h post-MCAO, the change rates of rCBF in different experimental groups were detected using the Perimed Laser Speckle Imager, and the representative pictures are presented in Figure 2E. As indicated in Figure 2F, the change rate of rCBF for the Model group was dramatically increased compared to that of the Sham group (*p* < 0.01), while that of the EGb761- and RR-treated groups was reduced compared to that of the Model group (*p* < 0.05 or *p* < 0.01), indicating that RR pretreatment significantly alleviated the reduced tendency of rCBF induced by MCAO and showing obvious dose dependence. 

### 3.5. RR Pretreatment Significantly Alleviated Neuronal Injury

HE staining was used to evaluate the extent of neuronal damage in the cortex and hippocampus. As indicated in Figure 2G, the neuronal cells of the cerebral cortex in the Sham group were intact with a large and rounded nucleus and clear nucleolar, the cells were distributed uniformly, and the outline was clear. In the Model group, the cell number of the neurons was reduced and the morphology of the cells was abnormal. Some neurons showed nuclear pyknosis, unclear nucleolar, and vacuoles of different sizes in the cytoplasm. As shown in Figure 2H, the pyramidal cells of the Sham group had about 3~4 layers in the hippocampus CA1 region, the cell was arranged neatly and closely, the structure of the cells was intact, with a large and round nucleus, and the nucleolar were visible. The cell number of the neurons of the hippocampus CA1 region was reduced and the morphology of the cell was abnormal in the Model group. Some neurons showed nuclear pyknosis, unclear nucleolar, and vacuoles of different sizes in the cytoplasm. As expected, the pathological manifestations of neuronal necrosis were reduced both in the EGb761 group and the RR pretreatment group in two lesion regions compared to those of the Model group, indicating that RR pretreatment significantly alleviated neuronal injury in the cortex and hippocampus. 

### 3.6. Changes in Gut Microbiota Composition Induced by RR

**Figure 3:**
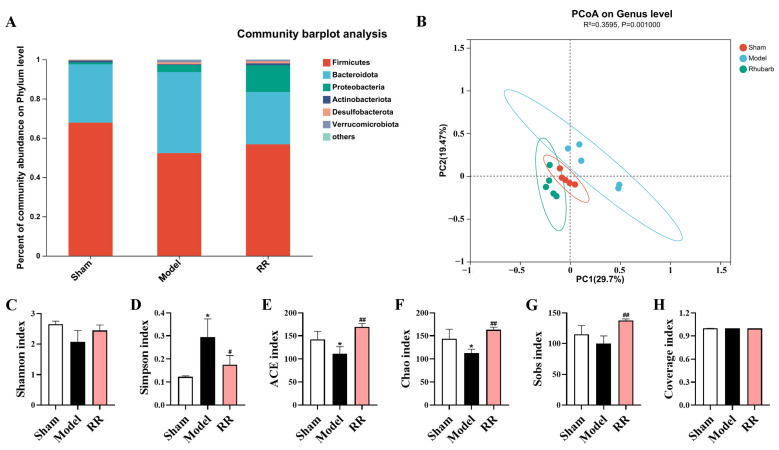
The results of microbial diversity analysis. (**A**) The top 10 microbial community composition of the phylum level. (**B**) The plot of the result of the principal coordinate analysis (PCOA) for β-diversity at the genus level. (**C**–**H**) The Shannon, Simpson, ACE, Chao, Sobs, and Coverage index analysis for α-diversity (values are expressed with mean ± SD; versus the Sham group: * *p* < 0.05; versus the Model group: ^#^ *p* < 0.05, ^##^ *p* < 0.01).

The 16S rRNA gene sequence was employed to assess the influence of RR on the intestinal microbiota in the MCAO rats. The top 10 species of relative abundance at the phylum level for each group were selected based on the species annotation results to produce a column-cumulative plot of the species’ relative abundance, and their proportion in each group was presented to compare the variance of species composition among the groups. At the phylum level, the proportion of *Firmicutes* to *Bacteroidota* was severely misaligned in the Model group compared to that in the Sham group: the *Firmicutes* reduced significantly, while the *Bacteroidota* increased significantly. Interestingly, RR pretreatment showed a tendency to bring the abundance of these two bacteria closer to that of the Sham group (Figure 3A). Shannon, Chao, ACE, Sobs, and Simpson indices were used to assess the microbial alpha diversity. The results demonstrated that the Simpson index increased significantly, and the Chao and ACE indices of the Model group decreased significantly compared to that in the Sham group, while RR pretreatment reversed these changes (*p* < 0.05 or *p* < 0.01, Figure 3C–G). The Coverage index was used to measure the probability that the species in the sample were detected, and the Coverage index of each sample library was above 99% in this study (Figure 3H). Principal coordinate analysis (PCOA) was used to assess the beta diversity of the microbes to assess the variation in the microbial composition between groups. The results of PCOA demonstrated that the Sham group and the Model group could be clearly distinguished, and the RR intervention tended to be closer to that of the Sham group (Figure 3B). 

**Figure 4:**
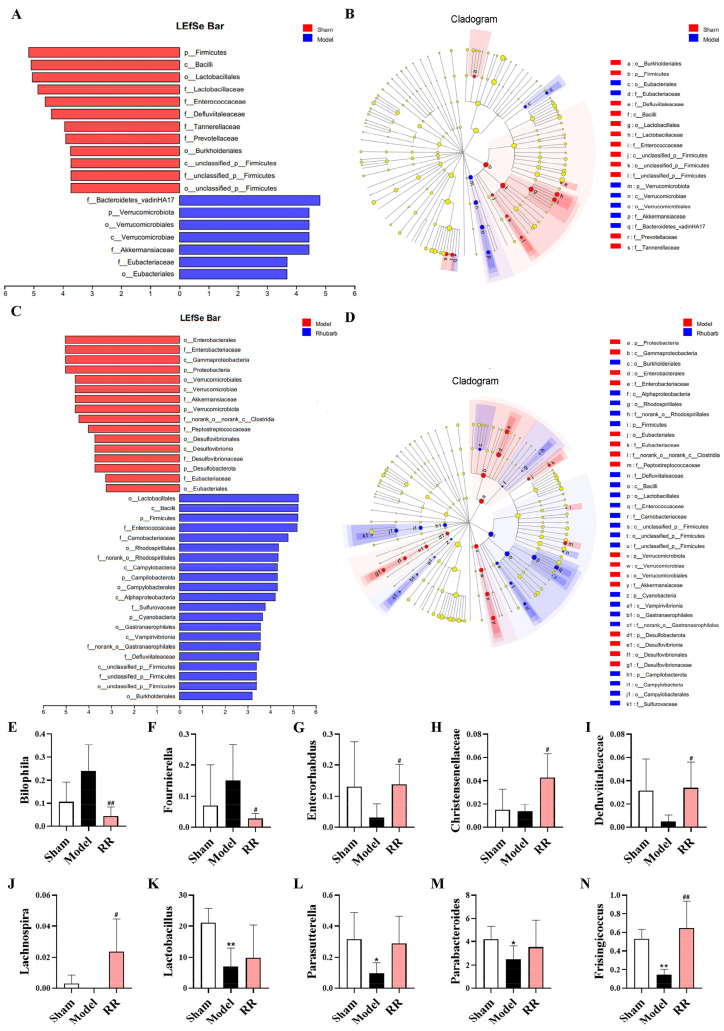
Results of the screening for differential microorganisms among groups. (**A**) The bar diagram of LEfSe analysis of the microbial community of Sham versus Model groups. (**B**) Taxonomic cladogram obtained by LEfSe analysis of Sham versus Model groups. (**C**) The bar diagram of LEfSe analysis of microbial community between the Model and RR groups. (**D**) Taxonomic cladogram obtained by LEfSe analysis for the Model and RR groups. (**E**–**N**) The relative abundance of *Bilophila*, *Fournierella*, *Enterorhabdus*, *Christensenellaceae*, *Defluviitaleaceae*, *Lachnospira*, *Lactobacillus*, *Parasutterella*, *Parabacteroides*, and *Frisingicoccus* in each group (values are expressed as mean ± SD; versus Sham: * *p* < 0.05, ** *p* < 0.01; versus Model: ^#^ *p* < 0.05, ^##^ *p* < 0.01).

The linear discriminant analysis effect size (LEfSe) analysis was employed to find the gut microbial markers with significant differences between groups. The microbial community composition in the Model group was dramatically changed versus that in the Sham group, which was characterized by a significant decrease in *Firmicutes*, *Lactobacillales*, *Enterococcaceae*, and *Defluviitaleaceae* (Figure 4A,B). Significant changes in the microbial community were found in the RR intervention group versus the Model group, which was characterized by a significant increase in *Firmicutes*, *Bacilli*, *Lactobacillales*, and *Enterococcaceae* (Figure 4C,D). In the Model group, the beneficial bacteria such as *Lactobacillus*, *Parabacteroides*, and *Parasutterella* were significantly decreased versus the Sham group, and the harmful bacteria such as *Bilophila* and *Fournierella* tended to be elevated, while the harmful bacteria such as *Fournierella* and *Bilophila* were dramatically reduced, and the beneficial bacteria such as *Enterorhabdus*, *Defluviitaleaceae*, *Christensenellaceae*, and *Lachnospira* were dramatically increased in the RR intervention group at the genus level (*p* < 0.05 or *p* < 0.01, Figure 4E–N).

### 3.7. H-NMR Analysis and Multivariate Analyses

**Figure 5:**
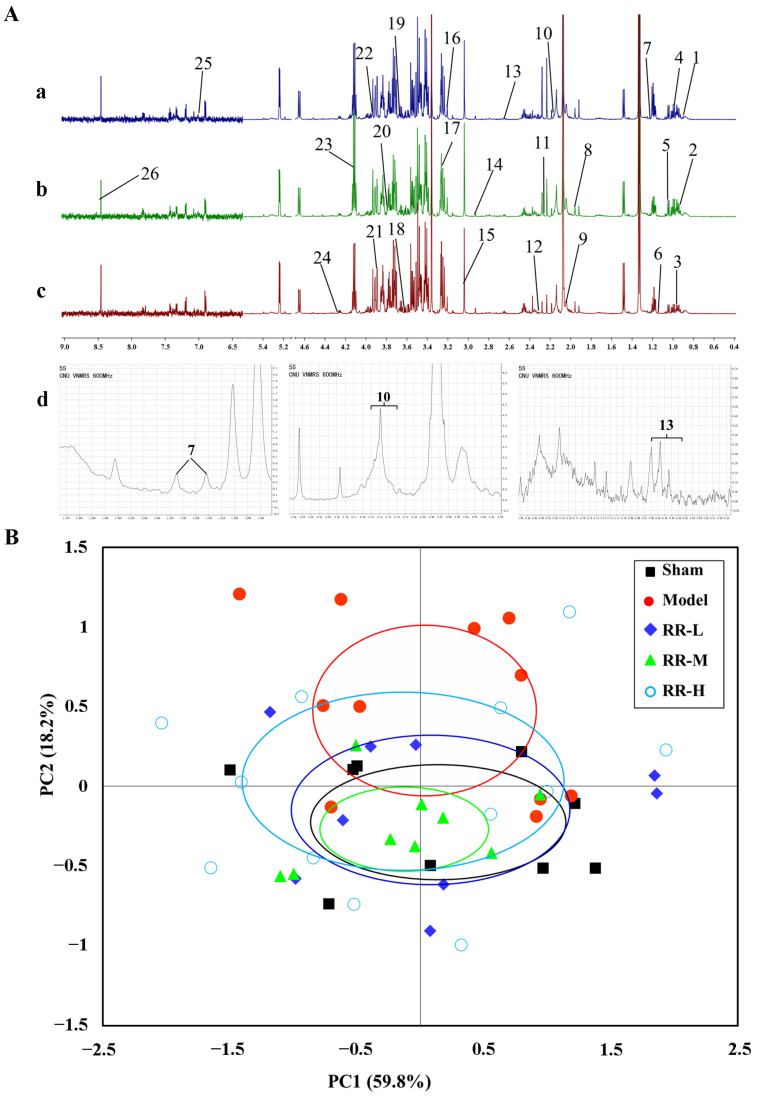
The representative spectra and the PCA score plot for metabolomics analysis. (**A**) The representative spectra of the RR-H group (**a**), Model group (**b**), and Sham group (**c**) from ^1^H-NMR. 1. 2-hydroxybutyrate, 2. isoleucine, 3. leucine, 4. valine, 5. 3-hydroxyisobutyrate, 6. propylene glycol, 7. 3-aminoisobutyric acid, 8. N6-acetyllysine, 9. lipid, 10. glutamine, 11. acetone, 12. acetoacetate, 13. methionine, 14. N, N-dimethylglycine, 15. creatine, 16. choline, 17. trimethylamine N-oxide, 18. myo-inositol, 19. ethanol, 20. glucose, 21. betaine, 22. serine, 23. lactate, 24. threonine, 25. histidine, 26. formate. (**d**) The enlarged nuclear magnetic resonance images of compounds 7, 10, and 13. (**B**) The PCA score plot for the serum metabolomics analysis of rats by the ^1^H-NMR.

The alterations in the serum metabolites were measured by the ^1^H-NMR spectroscopy analysis. The representative spectra of RR-H, Model, and Sham groups are shown in Figure 5A, and the changes in metabolism can be readily observed. Among them, the enlarged nuclear magnetic resonance images of compounds 7, 10, and 13 are shown in Figure 5A, and the identification of metabolites refers to the two published studies of G A Nagana Gowda et al. [34,35].

In this study, PCA was utilized to observe sample clustering, identify possible outlying samples, and reveal the systematic trends of the data based on the HR-MAS CPMG ^1^H NMR spectra. The data processing was carried out according to the literature method, and the center of each ellipse indicates the mean and the margin indicates one standard deviation [36,37,38]. The PCA score plot showed that the Sham group was separate from the Model group, while the RR treatment group partially overlapped with the Sham group, and the values were 59.8% for PC1 and 18.2% for PC2 (Figure 5B). Therefore, the PCA analysis showed that RR was able to regulate the metabolic changes induced by MCAO in the rat. 

**Figure 6:**
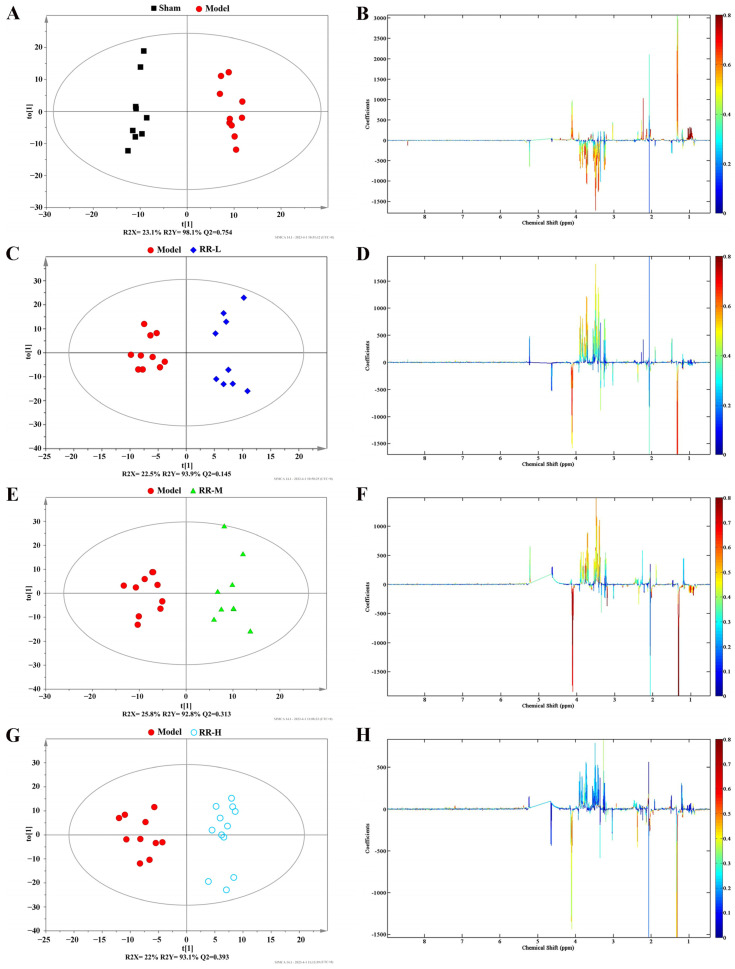
The OPLS-DA results for serum metabolomics. (**A**,**C**,**E**,**G**) The plots from OPLS-DA analysis for Model versus Sham, RR-L, RR-M, and RR-H groups. (**B**,**D**,**F**,**H**) The corresponding coefficient loading plots for Model versus Sham, RR-L, RR-M, and RR-H groups.

To further screen the differential metabolites from RR-treated serum samples, the supervised OPLS-DA model was employed to analyze the data, and the plots from the OPLS-DA analysis and corresponding coefficient loading are demonstrated in Figure 6. The OPLS-DA results showed significant separation between the Model group and the Sham group, and also the RR treatment groups and the Model group, in the serum samples. The parameters of descriptive (R2Y) and predictive characteristics (Q2), respectively, were 98.1% and 0.754 (Model vs. Sham group), 93.9% and 0.145 (RR-L vs. Model group), 92.8% and 0.313 (RR-M vs. Model group), and 93.1% and 0.393 (RR-H vs. Model group) of the OPLS-DA model, indicating the high explanation rate and a certain predictive value of the OPLS-DA model. 

Additionally, variations in metabolites in MCAO rats with or without RR intervention were visualized using color-coded coefficient plots. In Figure 6, significant changes in the Model vs. Sham groups or RR treatment vs. Model groups are shown (*p* < 0.05). 

**Figure 7:**
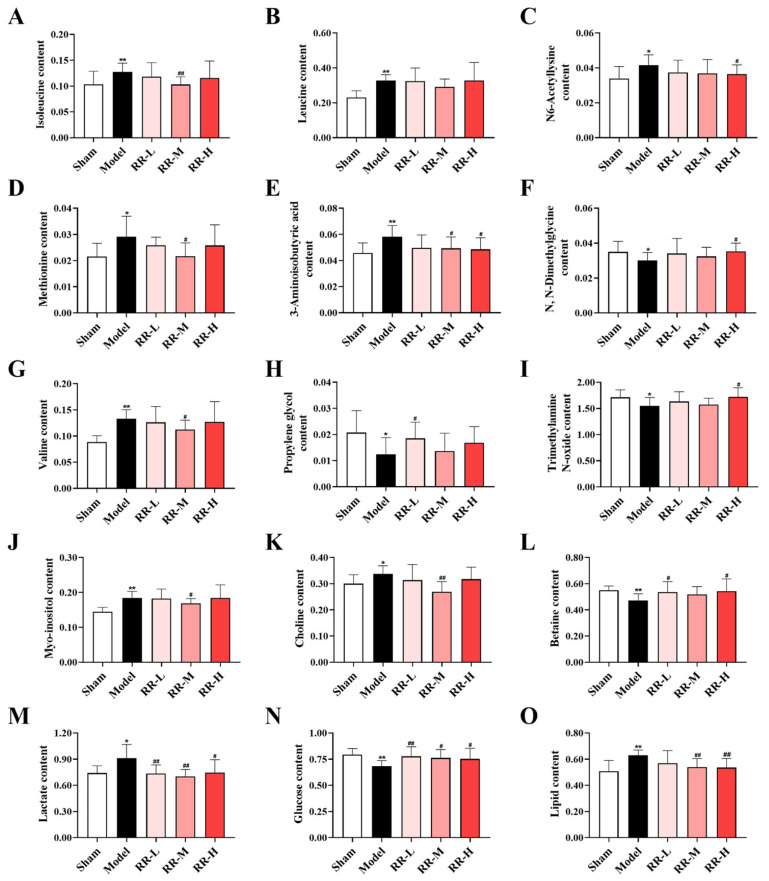
Potential metabolite changes in the serum of MCAO rats with the RR treatment: (**A**) isoleucine, (**B**) leucine, (**C**) N6-acetyllysine, (**D**) methionine, (**E**) 3-aminosobutyric acid, (**F**) N, N-dimethylglycine, (**G**) valine, (**H**) propylene glycol, (**I**) trimethylamine N-oxide, (**J**) myo-inositol, (**K**) choline, (**L**) betaine, (**M**) lactate, (**N**) glucose, (**O**) lipid (values are expressed with mean ± SD; versus the Sham group: * *p* < 0.05, ** *p* < 0.01; versus the Model group: ^#^ *p* < 0.05, ^##^ *p* < 0.01).

Then, VIP > 1 combined with *p*-value ≤ 0.05 was used as a basis for candidate metabolite selection. A total of 26 significantly changed metabolites (Model versus Sham) are summarized in Supplementary Table S2 (including the information from metabolites such as chemical shift, fold change, VIP value, and *p*-value). Additionally, 15 metabolites (including amino acid metabolites such as isoleucine, leucine, N6-acetyllysine, methionine, 3-aminoisobutyric acid, N, N-dimethylglycine, and valine; alcohol metabolites such as propylene glycol; amine metabolite trimethylamine N-oxide; inositides such as myo-inositol; organic base metabolites such as choline and betaine; organic acids such as lactate; and other metabolites, i.e., glucose and lipid), with significant changes both in Model rats and RR-treated rats, were considered to be potential metabolic biomarkers. As shown in Figure 7, the quantification analysis results of these candidate metabolites showed that the levels of isoleucine, lactate, valine, N6-acetyllysine, methionine, choline, myo-inositol, lipid, and 3-aminoisobutyric acid were significantly downregulated after treatment with RR, and the levels of propylene glycol, N, N-dimethylglycine, trimethylamine N-oxide, glucose, and betaine were significantly upregulated after treatment with RR (*p* < 0.05 or *p* < 0.01).

### 3.8. Metabolic Pathway Analysis

**Figure 8:**
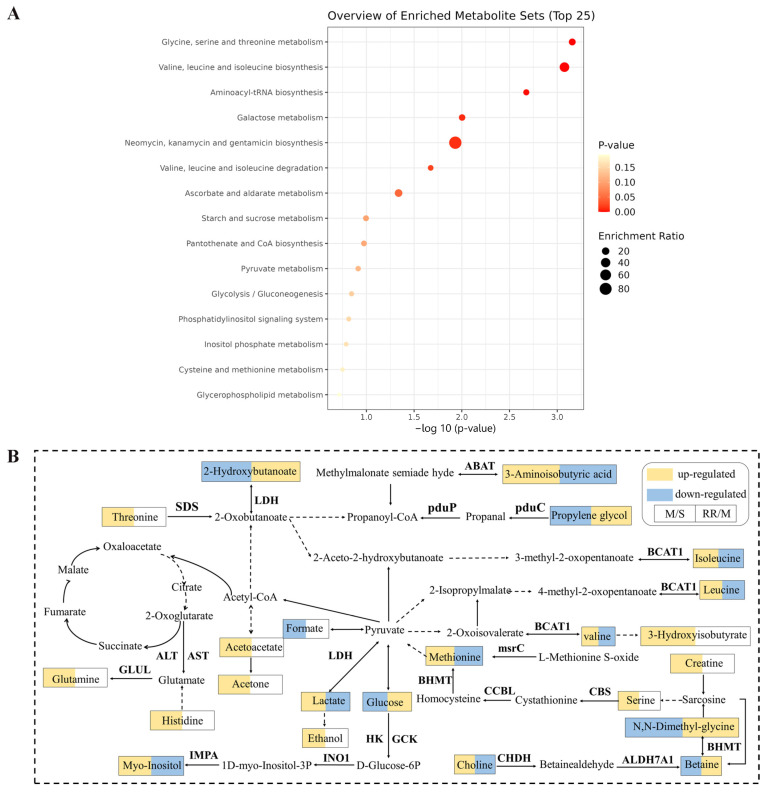
The results of metabolic pathway analysis. (**A**) The bubble plot: signaling pathways for the differential metabolites. (**B**) Relationship network diagram of differential metabolites.

To further analyze the effects of RR on metabolic pathways in MCAO rats, the obtained potential serum metabolic markers from rats were subjected to KEGG pathway enrichment analysis and the results showed that the differential metabolites in serum were enriched to 15 pathways, of which 7 pathways had significant differences, namely glycine, serine, and threonine metabolism; valine, leucine, and isoleucine biosynthesis; aminoacyl-tRNA biosynthesis; galactose metabolism; neomycin, kanamycin, and gentamicin biosynthesis; valine, leucine and isoleucine degradation; and ascorbate and aldarate metabolism (Figure 8A). The relationship network diagram of these related metabolites was plotted based on the KEGG database (Figure 8B).

### 3.9. Intestinal Microbes—Serum Metabolites Correlation Analysis

**Figure 9:**
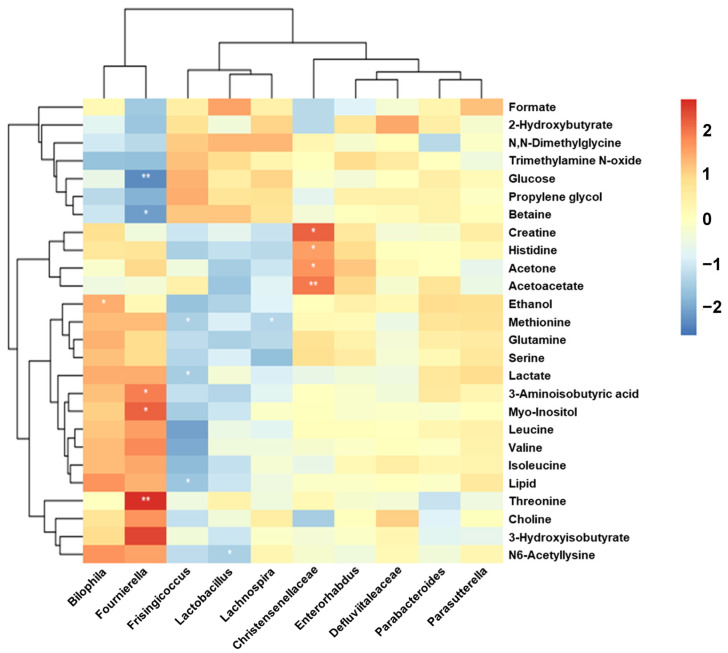
The heat map for the intestinal microbe–metabolite correlation analysis (red symbols for a positive relevance, whereas blue symbols for a negative relevance. The depth of color is positively related to the strength of correlation, * *p <* 0.05, ** *p <* 0.01).

To assess the relationship of intestinal microbes and serum metabolites, 26 differential metabolites and 10 differential microbes were selected for Spearman correlation analysis. The results showed that significant correlations were found between gut microbes and serum metabolites (Figure 9). Among them, *Fournierella* was positively correlated with threonine, myo-inositol, and 3-aminosobutyric acid, and negatively correlated with betaine and glucose; *Bilophila* was positively related to ethanol; *Frisingicoccus* was negatively related to lipid, lactate, and methionine; *Lactobacillus* was negatively related to N6-acetyllysine; *Lachnospira* was negatively related to methionine; and *Christensenellaceae* was positively related to creatine, histidine, acetone, and acetoacetate. The results indicated that the role of RR in IS was relevant to the relationship of gut microbes and serum metabolites.

## 4. Discussion

**Figure 10:**
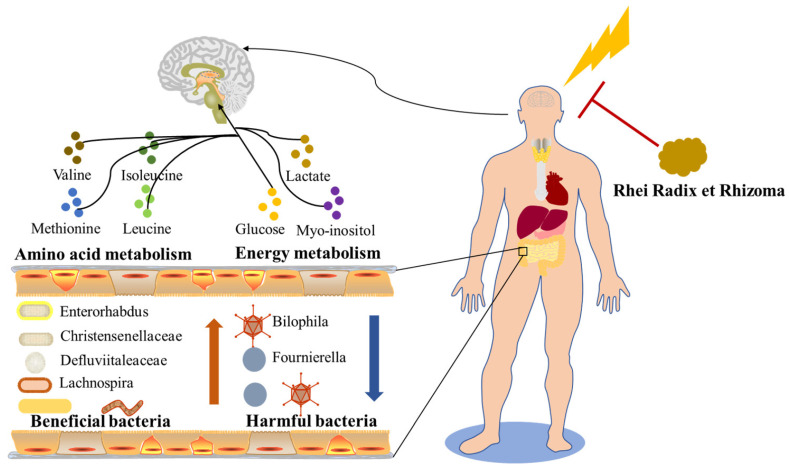
The pharmacological mechanism of RR in MCAO rats.

Our work revealed that RR dramatically improved the neurological function, cerebral infarction, cerebral edema, cerebral blood flow, and histopathological changes in MCAO rats, which presents obvious dose dependence. The harmful bacteria, for example, *Fournierella* and *Bilophila* tended to reduce, and the beneficial bacteria, for example, *Enterorhabdus*, *Defluviitaleaceae*, *Christensenellaceae*, and *Lachnospira* dramatically increased, after RR pretreatment. RR pretreatment significantly changed the levels of metabolites that were involved in the regulation of amino acid metabolism and energy metabolism (Figure 10).

RR is commonly used to treat acute IS based on Tong Fu’s theory of TCM. Previous studies have demonstrated that RR might exert a neuroprotective effect in cerebral ischemia by developing synaptic plasticity, inhibiting neuro-inflammation, and regulating the blood–brain barrier [16,17,39]. However, the mechanism of RR in the treatment of IS has not been completely elucidated due to the complexity of chemical components and the diversity of action targets of herbs. In our study, the major chemical components of the aqueous extract of RR were analyzed by the UHPLC-Q-Exactive MS system, and 92 chemical compositions were identified, consisting of 31 anthraquinones, accounting for about one-third of the total compounds. Among them, emodin, aloe emodin, rhein, chrysophanol, and physcion are the indexes for the quality control of RR in the Chinese Pharmacopoeia (CP). Modern pharmacological research has suggested that these anthraquinones have numerous pharmacology effects, for example, anti-inflammatory, antioxidant, immunity-regulating, and neuroprotective benefits, and it provides significant protection against disorders of the central nervous system, for example, IS, traumatic brain injury, and Alzheimer’s disease [40,41,42]. 

The MCAO model in rodents has been a reliable model for studying the mechanisms and potential therapeutic agents of cerebral ischemia in vivo because it is similar to human ischemia and has high reproducibility [43,44]. Two kinds of MCAO models are commonly used in research, the permanent MCAO model and the transient MCAO model, which simulate stroke patients who do not undergo or undergo vessel recanalization, respectively. In our study, a permanent MCAO model was applied because only 3% to 5% of patients could be treated with thrombolysis and only a few patients receive interventional therapy after thrombolysis failure [45,46]. We evaluated the role of RR pretreatment for IS from the perspective of neurological deficit score, cerebral infarct volume, cerebral edema rate, rCBF, and histopathological damage of the brain by establishing the permanent MCAO model of rats in this study. Meanwhile, EGb761, as an herbal extract with various pharmacological effects such as increasing rCBF and neuroprotection, was used as a natural neuroprotection drug for IS and was employed as a positive control drug to evaluate the efficacy of RR on IS in our study [47,48]. As a result, RR improved the neurological function, cerebral infarction, brain edema, and rCBF of rats at 24 h after MCAO, and reversed the pathological damage of brain tissue. Based on the above results, RR, especially RR-H, showed a significant preventive effect on IS.

IS develops as a result of complex pathomechanisms of brain injury caused by regional cerebral blood flow disorders, and its exact pathogenesis has not been fully elucidated. Gut microbes can affect the brain’s physical functions through the gut–brain axis, and exert an essential effect on the diseases of the central nervous system [49,50]. Lee et al. discovered that short-chain fatty acid (SCFA)-producing bacteria such as *Bifidobacterium longum*, *Clostridium symbiosum*, *Faecalibacterium prausnitzii*, and *Lactobacillus fermentum* can alleviate neurological damage and inflammatory responses after stroke [24]. Lian found that *Puerariae Lobatae* Radix-resistant starch (PLR-RS) alleviated brain damage caused by IS by increasing the abundance of *Akkermansia* and *Bifidobacterium*, and rescued intestinal microbiota imbalance [51]. At the phylum level, we found that *Firmicutes* was dramatically reduced and *Bacteroidota* was dramatically increased in the Model group compared to in the Sham group, consistent with the literature reports [52,53]. *Bacteroidota* and *Firmicutes* belong to the dominant phylum of intestinal microorganisms, accounting for more than 90% of intestinal microbes [54]. The majority members of *Firmicutes* are considered beneficial bacteria and the *Bacteroidota* are important opportunistic pathogens associated with inflammation, and the disorder of their proportion is considered to be closely related to IS [55,56]. Significant increases in the abundance of pathogenic bacteria or opportunistic bacteria such as *Eubacterium* and *Bacteroides* in gut microbes of MCAO rats have been reported, and pharmacological interventions may exert a therapeutic effect on IS by increasing the abundance of beneficial bacteria such as *Lachnospira* and *Akkermansia* [57]. We further noticed that the harmful bacteria such as *Fournierella* and *Bilophila* tended to be increased in MCAO rats, while the beneficial bacteria *Parabobacteroides* and *Lactobacillus* were significantly decreased compared to those in the Sham group, and the beneficial bacteria such as *Enterorhabdus*, *Defluviitaleaceae*, *Christensenellaceae*, and *Lachnospira* showed a decreasing trend in MCAO rats at the genus level. Among these, *Bilophila* is an opportunistic pathogen that can promote the inflammatory response by elevating the levels of serum TNF-α, IL-6, and amyloid A, and can affect metabolic pathways such as butyric acid and bile acid, which is highly associated with inflammatory bowel disease, IS, obesity, diabetes, and other inflammatory and metabolic diseases [58,59,60,61,62]. It was been found that the abundance of *Enterorhabdus* is associated with the microbial metabolites that were closely related to inflammation and neuroprotection after IS, and targeting *Enterorhabdus* may be a candidate therapy option for IS [56]. Therefore, the modulation of gut microbial composition might be an effective treatment for IS.

RR has been identified as having the effect of treating diseases by regulating intestinal microbiota [63,64,65,66]. The effect of RR on the intestinal microbiota of IS rats induced by MCAO was evaluated in our study. We found that the abundance of *Firmicutes* significantly increased and the *Bacteroidota* abundance decreased significantly in the RR pretreatment group, indicating that RR can improve the intestinal microbial composition of MCAO rats. At the genus level, we further observed a dramatic decrease in harmful bacteria such as *Fournierella* and *Bilophila*, and a dramatic increase in beneficial bacteria such as *Enterorhabdus*, *Defluviitaleaceae*, *Christensenellaceae*, and *Lachnospira* after RR pretreatment. These data suggest that RR prevents IS via regulating gut microbiota.

IS is also a disease related to metabolism. The metabonomic results demonstrated a significant increase in the levels of isoleucine, leucine, N6-acetyllysine, methionine, 3-aminoisobutyric acid, valine, myo-inositol, choline, lactate, and lipid in the serum samples of MCAO rats, and the levels of N, N-dimethylglycine, trimethylamine N-oxide, glucose, propylene glycol, and betaine were dramatically decreased compared to those of the Sham group in this study. As an energy-hungry organ, the brain is highly dependent on the energy provided by oxygen and glucose to maintain normal structure and function [67,68]. The metabolism of amino acids and energy are essential components of metabolomics and important pathogenesis of IS, in which the brain is under the condition of ischemia and hypoxia. The cellular energy metabolism changes from aerobic metabolism to anaerobic glycolysis after IS [69]. Glucose is metabolized to pyruvate and converted to lactic acid, and acidosis and tissue damage can be induced due to the substantial accumulation of lactic acid [69]. It was been reported that the serum lactic acid levels of IS patients were dramatically increased compared with the control group [70]. Isoleucine, leucine, and valine belong to branched-chain amino acids, and they were upregulated at the acute stage of IS patients [71]. Homocysteine, as the intermediate product of methionine metabolism, was considered to be an autonomous danger factor for stroke, and the serum methionine and homocysteine levels of patients with IS were significantly increased [72,73]. Choline is the essential intermediate metabolite of lipid metabolism, and also the precursor of acetylcholine and phosphatide choline, which can reflect the renewal of biofilm and the transmission status of neurotransmitters in vivo [74,75,76]. A significant increase in choline levels of blood in rats post-MCAO has been reported [77]. Hence, reversing the abnormal metabolites to normal levels could be an effective treatment for IS.

In our study, the metabonomic study results showed that RR pretreatment downregulated the levels of isoleucine, valine, 3-aminoisobutyric acid, N6-Acetyllysine, lipid, methionine, choline, lactate, and myo-inositol, and upregulated the N, N-dimethylglycine, trimethylamine N-oxide, glucose, propylene glycol, and betaine contents in the serum samples. The pathway enrichment results of differential metabolites revealed that RR may exert a preventive effect, mainly by modulating the synthesis and metabolism of amino acids and energy metabolism. These results are similar to those of previous studies. RR has been reported to regulate intracerebral hemorrhage and cholestasis by amino acids and energy metabolism [78,79]. Zhu et al. revealed that RR could reverse the abnormal lipid metabolism [80], and Zhang et al. demonstrated that RR extracts could reverse the metabolic abnormality in the urine of chronic kidney disease rats [81].

The intestinal microbiota is closely associated with the intestinal metabolites, which are the molecular basis of microbiota–host interactions [82]. The results of the Spearmen correlation analysis demonstrated a remarkable relevance between gut microbes and serum metabolites in our study, revealing that the impact of RR on IS was related to the interaction between intestinal microbes and serum metabolites. 

We selected the high-dose group of RR for the preliminary study of intestinal flora in this study. Future studies will be carried out on the effects of different doses of RR on intestinal flora and targeted metabolites in MCAO rats based on these results.

## 5. Conclusions

This research suggested a protective role of RR in the IS model rats induced by MCAO. The anti-IS mechanism of RR may be associated with the regulation of gut microbial composition and metabolite levels, which provides supporting data for further in-depth exploration of underlying mechanisms and the clinical application of RR.

## Data Availability

The original contributions presented in the study are included in the article and the Supplementary Materials. Further inquiries can be directed to the corresponding authors.

## Supplementary Materials

The following supporting information can be downloaded at: https://www.mdpi.com/article/10.3390/metabo14040216/s1, Table S1: Major chemical compounds of RR identified by UHPLC-Q-Exactive MS; Table S2: The list of identified metabolites from ^1^H-NMR spectra of the MCAO and RR treated rats’ serum samples. Supplementary Material: Supplementary experimental methods [33,83].

## Abbreviations

BSA, body surface area; CP, Chinese Pharmacopoeia; ddMS^2^, data-dependent acquisition; EGb761, ginkgo biloba extract; ESI, electrospray ionization; FT, Fourier transform; FIDs, free induction decays; HCD, higher-energy collision dissociation; HMDB, Human Metabolome Database; HESI, heated electrospray ionization source; IS, ischemic stroke; LEfSe, linear discriminant analysis effect size; MCAO, middle cerebral artery occlusion; MS, mass spectrometry; OPLS-DA, orthogonal partial least squares discriminant analysis; PCA, principal component analysis; PCOA, principal coordinate analysis; PCR, polymerase chain reaction; rt-PA, recombinant tissue plasminogen activator; rCBF, regional cerebral blood flow; ^1^H-NMR, ^1^H-nuclear magnetic resonance; TCM, Traditional Chinese medicine; TTC, 2,3,5-triphenyltetrazolium chloride; UHPLC, ultra-high-performance liquid chromatography; VIP, variable importance for projection.
