# Supplementary material for: The Water Extract of Rhubarb Prevents Ischemic Stroke by Regulating Gut Bacteria and Metabolic Pathways
Source: Metabolites. 2024 Apr 12;14(4):216. doi: 10.3390/metabo14040216 (PMC11052393; doi:10.3390/metabo14040216)
Supplement: Supplementary file 1 [file metabolites-14-00216-s001.zip › Supplementary Table S1.pdf]

Supplementary Table S1. Major chemical compounds of RR identified by UHPLC-Q-Exactive MS

NO.	*t*R/min	Formula	Measured mass (*m/z*)	Presumed compound
1	0.85	$C_5H_5N_5$	134.0474	Adenine
2	0.86	$C_6H_8O_7$	191.0201	Citric acid
3	1.07	$C_4H_6O_5$	133.0144	Malic acid
4	2.12	$C_{13}H_{16}O_{10}$	331.0677	Galloyl glucoside
5	2.15	$C_7H_6O_5$	169.0145	Gallic acid
6	2.58	$C_{19}H_{26}O_{15}$	493.1212	Gallic-O-di-glucoside
7	2.65	$C_9H_{11}NO_2$	164.072	Phenylalanine
8	5.03	$C_8H_8O_5$	183.0302	Methyl gallate
9	5.13	$C_{15}H_{18}O_8$	325.0938	P-coumaric acid-O-glucoside
10	5.83	$C_7H_6O_3$	137.0246	Salicylic acid
11	5.89	$C_{30}H_{26}O_{12}$	577.1365	Procyanidin B
12	5.89	$C_{15}H_{14}O_6$	289.0725	(+)-epicatechin
13	6.39	$C_{20}H_{20}O_{14}$	483.0787	Gallic acid-O-galloyl-glucoside
14	6.85	$C_{15}H_{14}O_6$	289.0724	(+)-Catechin
15	7.05	$C_{16}H_{20}O_9$	355.1052	Ferulic acid-O-glucoside
16	7.52	$C_{19}H_{22}O_9$	393.1207	6-Hydroxy-musizin-8-O-glucoside
17	8.29	$C_{27}H_{30}O_{15}$	593.1528	Emodin-O-di-glucoside
18	9.25	$C_{45}H_{38}O_{18}$	865.1944	Tri-catechin
19	9.48	$C_{20}H_{20}O_{12}$	451.0841	1-O-p-hydroxybenzoyl-6-O-galloyl-glucoside
20	9.69	$C_{22}H_{22}O_{12}$	477.1041	1-O-p-coumaryl-6-O-galloyl-glucoside/1-O-galloyl-6-O-p-coumaryl-glucoside
21	9.96	$C_{44}H_{34}O_{20}$	881.1594	ProcyanidinB-2,3,3'-di-O-gallate/procyandin B-5,3,3'-di-O-gallate
22	9.96	$C_9H_8O_3$	163.0403	P-Coumaric acid
23	10.13	$C_{23}H_{24}O_{13}$	507.1155	Ferulic acid-O-gallyl-glucoside

24	10.4	$C_{28}H_{28}O_{15}$	603.137	Catechin-O-gallioyl-Glucoside/epicatechin-O-gallioyl-glucoside
25	10.44	$C_{12}H_{10}O_5$	233.0462	2-methyl-5-carboxymethyl-7-hydroxychromogen
26	10.47	$C_{11}H_{10}O_3$	189.0561	2,5-dimethyl-7-hydroxychromone
27	10.93	$C_{21}H_{20}O_{10}$	431.099	Vitexin
28	10.98	$C_{27}H_{30}O_{16}$	609.1459	Rutinum
29	11.16	$C_{23}H_{26}O_{11}$	477.1415	Isolindleyin
30	11.22	$C_{44}H_{34}O_{20}$	881.1595	ProcyanidinB-2,3,3'-di-O-gallate/procyandin B-5,3,3'-di-O-gallate
31	11.25	$C_{42}H_{38}O_{20}$	861.1909	Sennoside B
32	11.41	$C_{21}H_{18}O_{11}$	445.0798	Rhein-8-O-glucoside
33	11.57	$C_{23}H_{26}O_{11}$	477.1421	Lindleyin
34	11.7	$C_{22}H_{18}O_{10}$	441.0834	(+) Catechin gallate/ (-) Epicatechin gallate
35	11.83	$C_{21}H_{22}O_9$	417.1192	Aloin/Isomer
36	11.92	$C_{37}H_{30}O_{16}$	729.1494	Procyanidin B-O-gallate
37	12.02	$C_{22}H_{22}O_{12}$	477.1034	1-O-p-coumaryl-6-O-galloyl-glucoside/1-O-galloyl-6-O-p-coumaryl-glucoside
38	12.05	$C_{15}H_{10}O_7$	303.0518	Quercetin
39	12.26	$C_{13}H_{14}O_4$	233.0824	2-(2'-hydroxypropyl)-5-methyl-7-hydroxychromone
40	12.33	$C_{42}H_{40}O_{19}$	847.2122	Sennoside C
41	12.33	$C_{27}H_{26}O_{12}$	541.1367	Resveratrol-4'-O-(6''-O-galloyl)-glucoside/resverantrol-4'-O-(2''-O-galloyl)-glucoside
42	12.5	$C_{15}H_{18}O_7$	309.0974	2-O-cinnamoyl-beta-D-glucose
43	12.56	$C_{29}H_{26}O_{16}$	629.1159	P-coumaric acid-O-di-galloyl-glucoside
44	12.76	$C_{42}H_{38}O_{20}$	861.1907	Sennoside A
45	12.79	$C_{13}H_{12}O_4$	231.0667	2-methyl-5-acetonyl-7-hydroxychromone
46	13.03	$C_{24}H_{22}O_{13}$	517.0998	Emodin-O-malony-glucoside/aloe-emodin-O-malony-glucoside

47	13.61	$C_{24}H_{22}O_{12}$	501.1051	Chysophanol-8-O-β-D-(6'-O-acetyl) glucoside
48	13.7	$C_{19}H_{22}O_9$	393.1202	Aloesone-O-glucoside
49	13.77	$C_{26}H_{28}O_{13}$	547.1451	2-(2' -hydroxypropyl)-5-methyl-7-hydroxytryptane-O-gallyl-glucoside
50	13.87	$C_{16}H_{12}O_6$	299.0566	Fallacinol
51	14.14	$C_{30}H_{30}O_{15}$	629.1538	4-(4'-hydroxyphenyl)-2-butanone-O-butanone-O-di-galloyl-glucoside
52	14.24	$C_{21}H_{20}O_{10}$	431.0991	Aloe-emodin-8-O-β-D-glucoside
53	14.24	$C_{22}H_{22}O_{11}$	461.1103	1-O-galloyl-2-O-cinnamoyl-β-D-glucoside
54	14.53	$C_{22}H_{20}O_{11}$	459.0938	Carboxyl chrysophanol-O-glucoside
55	15.21	$C_{42}H_{40}O_{19}$	847.2116	Sennoside D
56	15.45	$C_{42}H_{40}O_{18}$	831.2167	Chrysophanol (10-10') rhein type sennosides
57	15.66	$C_{23}H_{20}O_{12}$	487.0842	Rhein-O-acetyl-glucoside
58	15.93	$C_{20}H_{24}O_9$	407.1351	Torachrysone-8-O-glucoside
59	16.37	$C_{15}H_{12}O_6$	287.0566	Eriodictyol
60	16.51	$C_{23}H_{22}O_{11}$	473.1098	Emodin-O-acetyl-glucoside
61	16.8	$C_{21}H_{20}O_9$	415.103	Chrysophanol 8-O-glucoside/Chrysophanol 1-O-glucoside
62	17	$C_{45}H_{42}O_{21}$	917.2175	Chrysophanol (10-10') rhein type sennosides
63	17.03	$C_{26}H_{30}O_{10}$	501.1783	4-(4'-hydroxyphenyl) -2-butanone-O-feruloyl-glucoside
64	17.17	$C_{21}H_{20}O_{10}$	431.0992	Emodin-8-glucoside
65	17.26	$C_{21}H_{20}O_9$	415.1042	Chrysophanol 8-O-glucoside/Chrysophanol 1-O-glucoside
66	17.3	$C_{29}H_{26}O_{15}$	613.1213	Cinnamic acid-O-di-galloyl-glucoside
67	17.98	$C_{16}H_{10}O_7$	313.0362	Laccaic acid D
68	19.2	$C_{23}H_{22}O_{10}$	457.1133	Chrysophanol-O-acetyl-glucoside
69	19.26	$C_{22}H_{22}O_{10}$	445.1156	Physcion-8-O-β-D-glucoside

70	19.26	$C_{16}H_{12}O_5$	283.0618	Physcion
71	20.01	$C_{32}H_{32}O_{13}$	623.1789	4- (4' -hydroxyphenyl) -2-butanone-O-cinnamyl-gallyl-glucoside
72	20.01	$C_{15}H_{10}O_6$	285.0411	Citreorosein
73	20.11	$C_{23}H_{22}O_{11}$	473.11	Aloe-emodin-O-acetyl-glucoside
74	20.32	$C_{33}H_{34}O_{14}$	653.1899	4- (4 '-hydroxyphenyl) -2-butanone-O-feruloyl-gallyl-glucoside
75	21.11	$C_{24}H_{24}O_{11}$	487.1264	Physcion-O-acetyl-glucoside
76	21.11	$C_{16}H_{12}O_5$	283.0618	6-methyl-aloe-emodin
77	21.48	$C_{16}H_{10}O_6$	297.0411	6-methyl-rhein
78	22.76	$C_{33}H_{30}O_{13}$	633.1622	2, 5-dimethyl-7-hydroxytryptane-O-cinnamyl-gallyl-glucoside
79	22.79	$C_{15}H_{10}O_5$	269.0461	Aloe-emodin
80	23.62	$C_{32}H_{32}O_{12}$	607.1846	4-(4'-hydroxyphenyl)-2-butanone-4'-O-(2''-O-cinnamoyl-6''-O-galloyl)-glucoside
81	23.86	$C_{15}H_8O_6$	283.0255	Rhein
82	24.69	$C_{15}H_{12}O_4$	255.0669	Emodinanthrone
83	24.84	$C_{31}H_{26}O_{12}$	589.1365	1-methyl-8-hydroxy-9,10-anthraquinone-3-O-(6’-O-cinnamoyl)-glucoside
84	27.76	$C_{15}H_{12}O_3$	239.0719	Chrysophanol-9-anthrone
85	29.24	$C_{15}H_{10}O_5$	269.0461	Emodin
86	30.93	$C_{30}H_{20}O_9$	523.1045	Sennidin C
87	31.59	$C_{15}H_{10}O_6$	285.0411	Kaempferol
88	33.63	$C_{15}H_{10}O_4$	253.0512	Chrysophanol
89	33.63	$C_{30}H_{22}O_8$	509.125	Emodin bianthrone
90	0.87	$C_5H_5N_5$	136.0621	Adenine
91	1.33	$C_{10}H_{13}N_5O_4$	268.1043	Adenosine
92	1.46	$C_7H_6O_5$	171.0291	Gallic acid
93	4.47	$C_{30}H_{26}O_{12}$	579.1486	Procyanidin B
94	6.83	$C_{15}H_{14}O_6$	291.0871	(+)-Catechin

95	11.97	$C_{15}H_{14}O_4$	259.0969	Rhapontigenin
96	12.79	$C_{16}H_{10}O_7$	315.0505	Laccaic Acid D
97	14.55	$C_{16}H_{10}O_6$	299.0555	6-methyl-rhein
98	16.73	$C_{20}H_{24}O_9$	409.1498	Torachrysone 8-O-glucoside
99	17.25	$C_{21}H_{20}O_9$	439.1007	Chrysophanol 8-O-glucoside/Chrysophanol 1-O-glucoside
100	23.71	$C_{16}H_{12}O_5$	285.0762	Physcion
