# Supplementary material for: The Water Extract of Rhubarb Prevents Ischemic Stroke by Regulating Gut Bacteria and Metabolic Pathways
Source: Metabolites. 2024 Apr 12;14(4):216. doi: 10.3390/metabo14040216 (PMC11052393; doi:10.3390/metabo14040216)
Supplement: Supplementary file 1 [file metabolites-14-00216-s001.zip › Supplementary Table S2.pdf]

Supplementary Table S2. The list of identified metabolites from ^{1}H-NMR spectra of the MCAO and RR treated rats' serum samples.

No.	Metabolites	Chemical shift	Model VS Sham			RR-L VS MCAO			RR-M VS MCAO			RR-H VS MCAO		
		(ppm)(multiplicity)	VIP	FC	*p*-value	VIP	FC	*p*-value	VIP	FC	*p*-value	VIP	FC	*p*-value
1	2-Hydroxybutyrate	**0.90(t)**, 4.00(m)	1.29	0.67	0.046	1.21	1.19	0.081	<1	1.00	0.972	<1	1.06	0.567
2	Isoleucine	**0.94(t),** 1.01(d), 3.68(d)	1.52	1.25	0.005	1.32	0.95	0.558	1.93	0.81	0.001	1.02	0.92	0.324
3	Leucine	0.96(d), **0.97(d),** 1.718(m), 3.740(m)	2.15	1.44	0	1.15	0.99	0.882	1.86	0.89	0.05	<1	1.00	0.995
4	Valine	0.99(d), **1.05(d),** 2.281(m), 3.617(d)	2.23	1.48	0	1.07	0.98	0.793	1.48	0.86	0.015	<1	0.97	0.788
5	3-Hydroxyisobutyrate	**1.08(d)**	2.10	1.38	0	1.10	0.96	0.5	1.05	0.90	0.116	<1	0.94	0.595
6	Propylene glycol	**1.14(d)**	1.36	0.60	0.023	1.81	1.49	0.046	1.11	1.10	0.674	1.33	1.35	0.108
7	3-Aminoisobutyric acid	**1.23(d)，** 2.59(m)	1.83	1.27	0.004	1.87	0.85	0.057	1.54	0.85	0.038	2.03	0.84	0.016
8	N6-Acetyllysine	1.95(s)	1.53	1.23	0.014	1.40	0.90	0.162	1.07	0.88	0.134	1.72	0.88	0.036
9	Lipid	2.05	1.99	1.24	0	1.73	0.90	0.109	2.00	0.86	0.003	2.45	0.85	0.001
10	Glutamine	**2.16(m)**, 2.48(m)	1.77	1.18	0.003	2.48	0.92	0.074	<1	0.99	0.773	<1	0.93	0.235
11	Acetone	2.23(s)	1.80	2.04	0	1.21	1.05	0.882	<1	0.96	0.834	<1	0.97	0.895
12	Acetoacetate	2.29(s)	1.62	1.62	0.004	<1	0.98	0.921	<1	1.22	0.296	<1	0.92	0.62
13	Methionine	2.14(s)**, 2.67(m)**	1.43	1.35	0.023	1.06	0.89	0.228	1.58	0.74	0.025	<1	0.89	0.322

14	N, N-Dimethylglycine	2.93(s)	1.27	0.87	0.041	1.01	1.10	0.292	1.01	1.08	0.21	1.75	1.15	0.017
15	Creatine	3.05(s)	1.32	1.17	0.033	<1	0.92	0.343	<1	0.95	0.452	<1	0.95	0.504
16	Choline	3.21(s)	1.56	1.13	0.019	1.12	0.93	0.293	2.16	0.80	0	1.11	0.94	0.227
17	Trimethylamine N-oxide	3.27(s)	1.21	0.90	0.03	<1	1.05	0.305	<1	1.02	0.712	1.54	1.11	0.024
18	Myo-inositol	3.62(t)	2.10	1.25	0	1.23	0.97	0.63	1.23	0.89	0.02	<1	0.98	0.691
19	Ethanol	3.68(q)	1.64	1.10	0.013	1.26	0.95	0.334	1.15	0.94	0.13	<1	1.00	0.952
20	Glucose	5.24(d), 4.64(d), 3.88(dd),**3.79(m),** 3.48(m), 3.40(m), 3.24(dd)	1.94	0.86	0	1.94	1.14	0.01	1.46	1.12	0.017	1.31	1.10	0.049
21	Betaine	3.3(s), **3.91(s)**	1.80	0.86	0.001	1.65	1.14	0.042	1.25	1.10	0.075	1.44	1.15	0.037
22	Serine	**3.95(d),** 3.96(d), 3.986(d), 4.00(d)	1.59	1.12	0.004	<1	0.97	0.41	<1	1.00	0.99	<1	0.97	0.497
23	Lactate	1.33(d),**4.12(q)**	1.59	1.21	0.013	1.91	0.82	0.01	1.91	0.78	0.002	1.83	0.83	0.038
24	Threonine	1.32(d), **4.28(q)**	1.75	1.62	0.027	<1	0.80	0.254	<1	0.92	0.669	<1	0.87	0.4
25	Histidine	7.07(s)	1.23	1.24	0.033	<1	1.02	0.837	<1	0.97	0.751	<1	1.07	0.589
26	Formate	8.46(s)	1.85	0.63	0.001	<1	1.17	0.13	<1	1.06	0.553	<1	1.02	0.752
