# Supplementary material for: The Water Extract of Rhubarb Prevents Ischemic Stroke by Regulating Gut Bacteria and Metabolic Pathways
Source: Metabolites. 2024 Apr 12;14(4):216. doi: 10.3390/metabo14040216 (PMC11052393; doi:10.3390/metabo14040216)
Supplement: Supplementary file 1 [file metabolites-14-00216-s001.zip › Supplementary Material.pdf]

Supplementary Material

Supplementary Experimental Methods

S1. The dosage calculation of RR

The high dose of RR (RR-H) used in rats was calculated by the following formula based on the clinical dosage:

$$\text{Dosage of RR-H} = 15 \text{ g/person/day} \times 6.2/70 \text{ kg}$$

The maximum dose of RR was 15 g crude drug/day for adults in the clinic [1]. The ratio of the equivalent dose in humans and rats is 6.2, and the weight of the adult was considered as 70 kg.

S2. Gut microbiota determination and data analysis

The feces of the colon segment of rats were collected to analyze the intestinal microbial composition. The Genomic DNA of fecal simples were extracted according to the kit instructions and detected by 1% agarose gel electrophoresis. The specific barcode primers were designed, and the TransStart Fastpfu DNA Polymerase (Transgen Biotech, Beijing, China) was used for polymerase chain reaction (PCR) amplification. The 2% agarose gel electrophoresis and the QuantiFluorTM-ST blue fluorescence quantitative system (Promega, United States) were used to detect and quantify the PCR products, respectively. The kit of TruSeqTM DNA Sample Preparation (Illumina, United States) was utilized to construct the library for Illumina sequencing, and the sequencing was conducted on the Illumina platform. The PE reads obtained by Illumina sequencing were spliced based on the overlap relationship, and the quality control and filtering of the sequence was proceeded. The OUT cluster analysis of the samples at a 97% similarity level was carried out using the Uparse (version 7.0.1090, http://drive5.com/uparse/) and the microbial diversity indices were analyzed based on the results of the OTU cluster analysis.

The taxonomic analyses of the Silva (http://www.arb-silva.de) 16S rRNA database were carried out to classify the community species composition of each sample at each taxonomic level by applying the RDP classifier Bayesian algorithm (http://rdp.cme.msu.edu/), and the confidence threshold was set at 70%. The bioinformatics analysis was performed using the QIIME platform (http://qiime.org/scripts/assign_taxonomy.html).

S3. ^{1}H-NMR spectroscopy and NMR data analysis

3.1. Sample preparation of serum metabolisms

The blood from the abdominal aorta of rats was collected and centrifuged at 4 °C and 3000 rpm for 15 minutes to extract the supernatant, and then the serum sample was aspirated into 1.5 ml EP tubes. The 200 μL of serum was added into the deuterated phosphate buffer (NaH_2PO_4/K_2HPO_4, 0.045 mol/L, pH=7.47) of 400 μL, vortexed, and mixed, then centrifuged for 20 min at 4 °C and 12000 r/min, and the supernatant (about 550 μL) was transferred to an NMR tube of 5 mm for future analysis.

3.2. Nuclear magnetic resonance measurements and data processing

A 600 MHZ NMR spectrometer (Varian Inc, Palo Alto, Calif) equipped with a 5 mm inverse-proton triple resonance probe was employed to record the ^{1}H-NMR spectra of the serum samples at 599.672 MHz and 298 K. The relevant measurement parameters and data processing before principal component analysis (PCA) and orthogonal partial least squares discriminant analysis were carried out by referring to the literature (OPLS-DA) [2]. The chemical shift of the NMR spectra was corrected by the lactate and was referenced at δ 1.33.

3.3. Metabolites identification and multivariate statistical analysis

The online Human Metabolome Database (HMDB) (http://www.hmdb.ca/) and relevant published references were employed to identify the metabolites. The statistical analysis of the integral area of the metabolites was performed between the groups using the R software package, the clustering analysis of the samples was performed by PCA to reveal trends between the groups of samples, and the degree of separation between the groups of samples was visualized through the OPLS-DA. The OPLS-DA models were validated by replacing the response values of the tests and calculating R^2 (the total explained variation, related to the prediction accuracy of the model) and Q^2 (the predictive capacity, related to the fitting ability of the model), and the number of model validations was 200.

A color-coded coefficient map was produced to visualize and filter the variables that impact the model. The value of variable importance for projection (VIP) was used to screen for compounds with significant contributions to the classification of metabolites. The comparisons between groups were analyzed using the *t*-test, and the method of Benjamini and Hochberg was applied to adjust for the p-values. The metabolites were considered to be differential metabolites with the *p*-value $\leqslant$ 0.05 and VIP >1. The pathway enrichment analysis was performed by applying the Metaboanalyst platform (https://www.metaboanalyst.ca/) with differential metabolites, and the KEGG online database (https://www.genome.jp/kegg/) was used to analyze the significantly perturbed pathways of the differential metabolites.

References

1. State Pharmacopoeia Commission. *Pharmacopoeia of the People's Republic of China*, Part I. China Pharmacopoeia Science and Technology Press: Beijing, China, **2020**; 24.

2. Gao, J.; Bai, P.; Li, Y.Y.; Li, J.Z.; Jia, C.X.; Wang, T.S.; Zhao, H.B.; Si, Y.C.; Chen, J.X. Metabolomic profiling of the synergistic effects of ginsenoside Rg1 in combination with neural stem cell transplantation in ischemic stroke rats. *J. Proteome. Res.* **2020**, 19, 2676-2688. https: //doi.org/10.1021/acs.jproteome.9b00639.
